# Supplementary material for: Multicentre Evaluation of Hepika Test Clinical Accuracy in Diagnosing HPV-Induced Cancer and Precancerous Lesions of the Uterine Cervix
Source: Diagnostics (Basel). 2021 Mar 30;11(4):619. doi: 10.3390/diagnostics11040619 (PMC8066214; doi:10.3390/diagnostics11040619)
Supplement: Supplementary file 1 [file diagnostics-11-00619-s001.pdf]

Supplementary Materials:

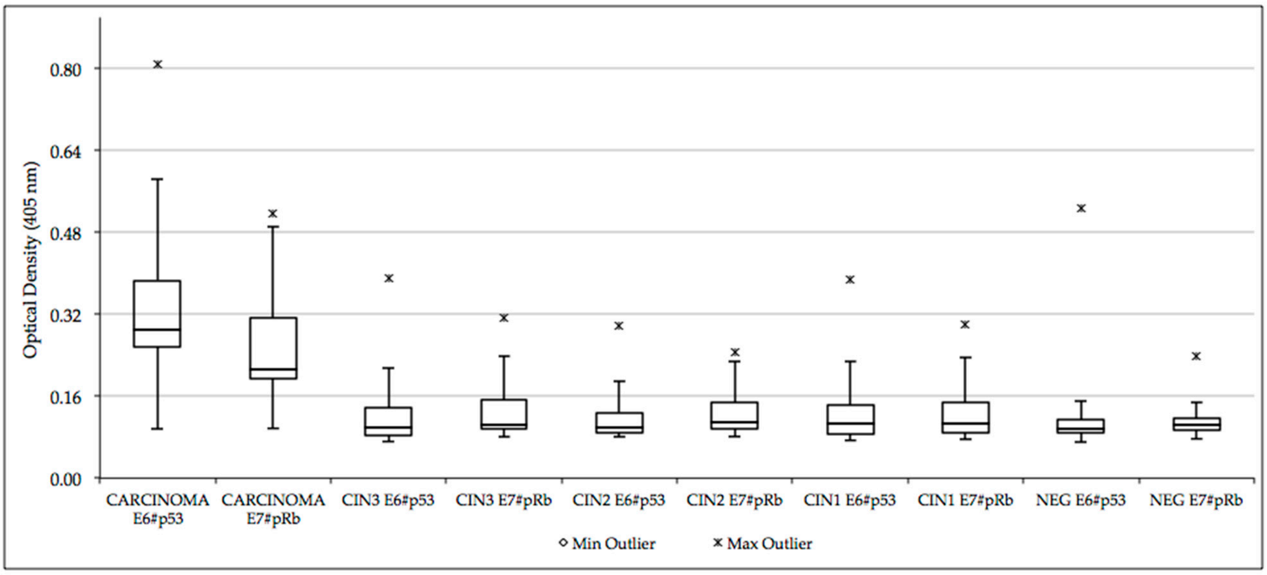

Figure 7. Distribution of complexes E6#p53 and E7#pRb optical density in relation to histology.
Note: The top of box represents the upper quartile (p75), bottom the lower quartile (p25), and the line the median (p50). The upper whisker extends to the largest point of the inter-quartile range from the upper quartile. The lower whisker extends to the smallest point of the inter-quartile range from the lower quartile. The outliers are plotted as individual points for each lesion grade.
